# Combinatorial Effects of Ursodeoxycholic Acid and Antibiotic in Combating *Staphylococcus aureus* Biofilm: The Roles of ROS and Virulence Factors

**DOI:** 10.3390/microorganisms12101956

**Published:** 2024-09-27

**Authors:** Anuradha Tyagi, Vinay Kumar, Navneet Joshi, Harish Kumar Dhingra

**Affiliations:** 1 Department of Biosciences, School of Liberal Arts and Sciences, Mody University of Science and Technology, Lakshmangarh 332311, Rajasthan, India; anuradhatyagi07@gmail.com (A.T.); navybiotech@gmail.com (N.J.); 2 Department of Medicine, Pennsylvania State University, Hershey Medical Center, Hershey, PA 17033, USA

**Keywords:** antibiotics, biofilm, combination therapy, *S. aureus*, ursodeoxycholic acid

## Abstract

*Staphylococcus aureus* is a biofilm-forming bacterium responsible for various human infections, one particularly challenging to treat due to its antibiotic resistance. Biofilms can form on both soft tissues and medical devices, leading to persistent and hard-to-treat infections. Combining multiple antimicrobials is a potential approach to overcoming this resistance. This study explored the effects of ursodeoxycholic acid (UDCA) combined with the antibiotic ciprofloxacin against *S. aureus* biofilms, aiming to evaluate any synergistic effects. Results showed that UDCA and ciprofloxacin co-treatment significantly reduced biofilm formation and disrupted pre-formed biofilms more effectively than either agent alone (*p* < 0.01). The combination also displayed a slight synergistic effect, with a fractional inhibitory concentration of 0.65. Additionally, the treatment reduced the production of extracellular polymeric substances, increased reactive oxygen species production, decreased metabolic activity, altered cell membrane permeability, and lowered cell surface hydrophobicity in *S. aureus*. Furthermore, it diminished biofilm-associated pathogenic factors, including proteolytic activity and staphyloxanthin production. Overall, the UDCA–ciprofloxacin combination shows considerable promise as a strategy to combat infections related to staphylococcal biofilms, offering a potential solution to the healthcare challenges posed by antibiotic-resistant *S. aureus*.

## 1. Introduction

Microorganisms have the ability to exist in two separate states inside natural environments: the planktonic form and the sessile form, i.e., as biofilm [1]. Several studies have reported the existence of biofilm on the surface of implanted medical devices and catheters along with biological tissues, leading to chronic diseases [2,3,4]. In humans, biofilms are responsible for up to 80% of all microbial infections, as reported by the National Institute of Health [5,6,7,8]. Microbial biofilm formation is facilitated by the production of a slimy coating called extracellular polymeric substance (EPS) [9]. EPS aids in cohesion of the bacterial community, anchoring bacterial cells to solid surfaces, and ensuring adequate hydration and nutrition availability. Apart from EPS, hydrophobicity of the cell surface and the presence of flagella and fimbriae also influence the attachment of the microbial cell to the substratum [10]. The extracellular matrix is composed of several macromolecules, such as extracellular polysaccharides, diverse lipids and proteins, poly-N-acetyl glucosamine, and extracellular DNA [11]. Biofilm offers a significantly enhanced protective habitat for bacteria to flourish, providing them with 10–1000 times more protection [1]. Additionally, it poses a challenge for external antimicrobial treatments since it is resistant to removal. Consequently, biofilm development is a fundamental mechanism for antibiotic resistance in pathogenic bacteria [12]. The global public-health issue of biofilm-mediated pathogenesis and its antibiotic-resistance traits necessitates the management of this health concern.

*Staphylococcus aureus* is a Gram-positive bacterial species that can be recognized both as a commensal bacterium and as a human pathogen. Reports indicate that around 30% of the human population is colonized with *S. aureus* [13], which can cause infective endocarditis and bacteremia, as well as infections in the skin, the soft tissues, the pleuropulmonary system, and medical devices. It is one of many bacteria capable of forming biofilms. *S. aureus* has been associated with a range of systemic diseases presenting substantial risks to the host’s life. The formation of biofilm necessitates a multiplex interaction among the bacterium, the extracellular matrix, and the immune response of the host. Bacteria can endure and induce persistent infections by exploiting the extracellular matrix to shield themselves from antimicrobial treatments and the immunological responses of the host [14]. During the maturation of biofilms, *S. aureus* has the ability to generate a variety of virulence factors, such as toxins and enzymes, which play a role in propagating the infection [15]. *S. aureus*’s capacity to create biofilm provides it with heightened resistance to antibiotics, posing a severe therapeutic obstacle [16]. Therefore, it is crucial to explore new and efficient therapeutic approaches to prevent the harmful effects of *S. aureus* biofilms. Multiple natural and synthesized substances have been utilized to fight against the infectious activity of *S. aureus* [17].

Currently, the research and development of new antibacterial compounds and antibiotic adjuvants have emerged as useful strategies to bolster the effectiveness of antibiotics in combating bacterial resistance. However, as compared to the expensive and unpredictable process of developing novel antimicrobial drugs, choosing natural chemicals as antibiotic adjuvants is seen as a secure, practical, and encouraging approach. For instance, our previous study has demonstrated the antimicrobial activity of ursodeoxycholic acid (UDCA) against biofilm-forming microbes [18]. UDCA (3α,7β-dihydroxy-5β-cholanic acid) is a type of secondary bile acid that is produced by dehydroxylation of primary bile acids in the gut [19]. It is mainly utilized for liver disorders; however, its role in treating other diseases such as neurodegenerative diseases, cancer, and obesity has also been explored [20]. Furthermore, it has been shown that UDCA plays a vital role in preserving a healthy equilibrium of gut bacteria by inhibiting the proliferation of harmful bacteria [21].

Despite having a plethora of biological activities, an antimicrobial may sometimes require synergistic action to fight against the rapidly evolving microbial community. Scientific reports have even recommended the better therapeutic efficacy achieved during a combined treatment using two compounds [22]. Therefore, this study was conducted to assess the impact of UDCA in conjunction with a conventional antibiotic, specifically, ciprofloxacin (a quinolone), on the formation of biofilms by *S. aureus*.

## 2. Materials and Methods

### 2.1. Chemicals, Bacterial Strain, and Culture Conditions

The strain of *S. aureus* (ATCC 25923) utilized in the study was acquired from the American Type Culture Collection, Manassas, VA, USA. It was stored in stock cultures at a temperature of −20 °C. The bacterial cultures were cultivated in brain–heart infusion broth in a shaker incubator overnight at 37 °C for all investigations, which were conducted in a laboratory setting. Anant Pharmaceuticals Pvt. Ltd., Ambernath, India, supplied the ursodeoxycholic acid. Ciprofloxacin and dimethyl sulfoxide (DMSO) were acquired from Sigma-Aldrich, St. Louis, MO, USA. The microbiological culture medium and agar were procured from Titan Biotech Ltd., Delhi, India.

### 2.2. Minimum Inhibitory Concentration (MIC) Quantification

The minimum inhibitory concentration (MIC) of UDCA and ciprofloxacin was determined individually against *S. aureus* using the conventional broth microdilution technique [23]. Briefly, two-fold serial dilutions of each compound were added to the wells of sterile 96-well plates containing brain–heart infusion broth (100 µL) with a bacterial concentration of 10^6^ CFU/mL. Both, UDCA and ciprofloxacin were dissolved in DMSO and added to the wells. Similarly, ciprofloxacin was dissolved in DMSO. Following a 24 h incubation period at 37 °C, the minimum inhibitory MIC was identified as the lowest concentration that completely halted the growth of the bacteria. This was performed by measuring the optical density (OD) at 600 nm using a microplate reader (Power Wave XS 2, BioTek, Winooski, VT, USA).

### 2.3. Fractional Inhibitory Concentration (FIC) Index Determination

The fractional inhibitory concentration index was determined using the checkerboard method to assess the interaction between UDCA and ciprofloxacin against *S. aureus* [24]. In summary, a culture of *S. aureus* cells that had been cultivated overnight (10^6^ CFU/mL) was placed into 96-well plate tubes containing sterilized growth media (with a volume of 200 µL). In addition, various quantities of UDCA (40.625, 81.2, 162.5, 325, 650, and 1300 μg/mL) and ciprofloxacin (0.05, 0.1, 0.2, 0.3, 0.4, 0.5 µg/mL) were separately and in combination were administered to the wells and incubated further at 37 °C for 24 h. The FIC index was calculated as per the formula:

FIC index = FIC of A + FIC of B.

Additionally, FIC of each test compound was calculated according to the formula:

FIC of A = MIC of A in combination∕MIC of A alone and FIC of B = MIC of B in combination/MIC of B alone.

FIC index < 0.5 indicates synergistic effect, 0.5–0.75 indicates partial synergistic effect, 0.75–1 indicates additive effect, 1-4 indicates no effect and >4 indicates antagonistic effect.

### 2.4. Effect on Biofilm Formation

The crystal violet assay was used to examine the impact of different treatments on the formation of staphylococcal biofilms. In summary, a culture of *S. aureus* was diluted with brain–heart infusion broth containing 2% sucrose to obtain a bacterial suspension of 10^6^ cells/mL. Subsequently, 100 µL of bacterial suspension was added to 96-well plates, along with a specific combination of UDCA (325 µg/mL) and ciprofloxacin (0.2 µg/mL). The plates were then incubated at a constant temperature of 37 °C for 24 h. Following incubation, the non-adherent bacteria were washed off using sterile distilled water, repeating the process thrice. Thereafter, the biofilm was stained using a 0.1% crystal violet solution at room temperature for a duration of 15 min. After removal of excess dye from the 96-well plate, the plate was rinsed three times with sterile distilled water. The biofilm stained with crystal violet was dissolved in 95% ethanol and then dehydrated at a temperature of 55 °C for a duration of 10 min. The biofilm was quantified by measuring its absorbance at a wavelength of 570 nm. Additionally, the effect of test-compound combination was also analyzed qualitatively on biofilm structure. *S. aureus* (10^6^ cells/mL) were added along with fresh culture medium containing sterilized cover slips in a 6-well plate. Subsequently, a mixture of UDCA and ciprofloxacin was introduced into the wells, along with the separate components alone, and left to incubate for 24 h at 37 °C. Following the incubation period, the cover slips were washed with distilled water to eliminate any cells that were not firmly attached. The biofilms that attached to the coverslips were subsequently dyed using a 0.1% crystal violet solution and observed using a light microscope (Olympus, Tokyo, Japan) at a magnification of 200×.

### 2.5. Effect on Biofilm Eradication

To evaluate the impact of selected doses and combination of the test compounds (UDCA- 325 µg/mL and ciprofloxacin- 0.2 µg/mL on eradication of preformed biofilm of *S. aureus*, crystal violet assay was used. An overnight culture of *S. aureus* adjusted to 10^6^ cells/mL using brain–heart infusion broth containing 2% sucrose was inoculated into a 96-well plate and kept for incubation at 37 °C for 24 h for biofilm formation. Following incubation, the free-floating cells were removed from each well by aspiration, and the attached cells were rinsed with sterile broth (150 μL). Subsequently, the chosen mixture of UDCA and ciprofloxacin was introduced into the wells and incubated at 37 °C for 24 h. Subsequently, the planktonic bacteria were thoroughly washed thrice, using sterile distilled water. The biofilm was stained using a 0.1% crystal violet solution at room temperature for a duration of 15 min. After removing the extra dye from the 96-well plates, the plates were washed three times with sterile distilled water. The crystal violet-stained biofilm was dissolved in 95% ethanol and subsequently dehydrated at 55 °C for 10 min. The biofilm was quantified by measuring its absorbance at a wavelength of 570 nm.

### 2.6. Quantification of Total Biofilm Protein

To quantify the total protein content of the biofilm produced by *S. aureus*, the bacterial culture grown overnight was diluted to a concentration of 10^6^ CFU/mL. It was next cultivated in 5 mL of sterilized growth media that had been enriched with specific amounts of UDCA (325 µg/mL) and ciprofloxacin (0.2 µg/mL). After it was incubated for 24 h at a temperature of 37 °C, the planktonic organisms were removed, and the tubes were then washed with Milli-Q water. Subsequently, 5 mL of aseptic 0.3 M NaOH solution was introduced into each tube and kept at a temperature of 100 °C, for a duration of 30 min. After boiling, the samples were subjected to centrifugation at a speed of 8000 rpm for 10 min. The protein concentration in the supernatant was quantified using the Bradford technique.

### 2.7. Measurement of Extracellular Polymeric Substance (EPS)

To assess the impact of the combined test chemicals on EPS generation, the phenol-sulfuric acid technique was employed, following the protocol outlined in Reference [25]. In brief, bacterial culture was diluted in brain–heart infusion broth to a concentration of 10^6^ CFU/mL. Afterwards, it was mixed with the test compounds (UDCA 325 µg/mL and ciprofloxacin 0.2 µg/mL) and incubated at 37 °C for 24 h. After incubation, the cell suspensions were centrifuged at a speed of 8000× *g* for 10 min. Thereafter, 1 mL of 1.5 M NaCl (1 mL) was introduced. Subsequently, the cell suspension underwent centrifugation for 10 min at 5000× *g*. Following this, the resultant supernatants (60 μL) were mixed with 5% phenol (60 μL) and sulfuric acid (4 mL) and again incubated at 30 °C for 10 min. The absorbance was quantified at a wavelength of 490 nm following the incubation period. The calculation for the creation of EPS (%) was derived using the following equation:

EPS quantification (%) = (OD_treatment_/OD_control_) × 100

### 2.8. Estimation of Cellular Reactive Oxygen Species (ROS)

The production of reactive oxygen species in the microorganism was evaluated under different conditions, namely, with or without the specific combinations of UDCA and ciprofloxacin, using the methodology described elsewhere [26]. The experiment was carried out with 96-well polystyrene microplates. The microbial cell concentration was adjusted to 10^6^ cells/mL and then exposed to the combination of test compounds (UDCA 162.5 µg/mL and ciprofloxacin 0.2 µg/mL). The mixture was incubated for 2 h at 37 °C. Subsequently, the cells were rinsed with phosphate buffer saline and resuspended with a concentration of 10 µM H2DCFDA. They were then allowed to incubate at 37 °C for 1 h. Afterwards, the cells that had been stained were rinsed with PBS and the amount of fluorescence was measured using a spectrofluorimeter (Perkin Elmer, Waltham, MA, USA) with excitation and emission wavelengths of 485 nm and 535 nm being determined, respectively.

### 2.9. Cell Membrane Permeability Estimation

The effect of the combined test compounds on bacterial cell membrane permeability was assessed by utilizing the ethidium bromide influx method [27]. *S. aureus* cells (10^6^ CFU/mL) were taken and either exposed to the combination of UDCA and ciprofloxacin or prepared alone at 37 °C for 3 h. Subsequently, a solution of ethidium bromide (0.5 μg/mL) was added to all the experimental groups and incubated for an additional 30 min. After the incubation period, the amount of fluorescence was measured using a fluorimeter (Perkin Elmer, Waltham, MA, USA) with excitation and emission wavelengths of 520 nm and 590 nm being determined, respectively.

### 2.10. Measurement of Induced Cell Autolysis

The effect of the combined UDCA and ciprofloxacin on the induced autolysis of *S. aureus* was investigated using the previously described experimental technique [15]. *S. aureus* cells at a concentration of 10^6^ CFU/mL were added to growth media containing sodium chloride (1 M). UDCA was introduced to the tubes at a concentration of 325 μg/mL, while ciprofloxacin was added at a concentration of 0.2 µg/mL. These substances were added either separately or together. The tubes were then incubated at 37 °C until the optical density of the bacterial culture reached 0.7. After the incubation period, the cell suspensions from each experimental group were subjected to centrifugation at 8000 rpm for 5 min. After being subjected to centrifugation, the cell suspensions were rinsed with Milli-Q water. The cell pellets were dissolved in the autolysis buffer, which included 0.1% Triton X-100 and 50 mM Tris–HCl. The bacterium’s autolysis was assessed at a wavelength of 580 nm, with measurements taken every 30 min, for a total duration of 1 h.

### 2.11. Measurement of Cell Surface Hydrophobicity

The alteration in the hydrophobicity of the cell surface of *S. aureus* was evaluated by employing the bacterial adherence to hydrocarbon assay [28]. Initially, *S. aureus* culture (10^6^ CFU/mL) was treated with or without the test compounds (UDCA and ciprofloxacin). After incubation for 24 h at 37 °C, the cells were collected by centrifugation at 8000 rpm for 10 min. Subsequently, the gathered cells were solubilized in 3.4 mL of Milli-Q water and ultimately, the optical density was measured at 420 nm using a spectrophotometer (initial OD) (Power Wave XS 2, BioTek, Winooski, VT, USA). Subsequently, 0.6 mL of chloroform was added to each set and incubated for a further 30 min. Afterwards, the optical density (OD) of the upper layer was measured at a wavelength of 420 nm, yielding the final OD value. Cell surface hydrophobicity was estimated by the following formula:

Cell surface hydrophobicity (%) = {(initial OD_420nm_ − final OD_420nm_)∕initial OD_420nm_ × 10

### 2.12. Analysis of Cellular Auto-Aggregation

The auto-aggregation pattern of *S. aureus* was determined by using the methodology described elsewhere [15]. In summary, *S. aureus* was cultivated in sterilized growth media with or without specific compound combinations (UDCA and ciprofloxacin) for 24 h at 37 °C. In addition, centrifugation was performed at 8000 rpm for 10 min in order to gather the cell pellet. Subsequently, the cell pellets were re-suspended in 5 mL of sterile PBS to create a cell suspension, which was then incubated at 37 °C for 20 h. Thereafter, the cell auto-aggregation was evaluated by measuring the optical density (OD) of the upper part of the suspension at a wavelength of 630 nm.

### 2.13. Effect of on the Metabolic Profile

The assessment of the metabolic activity of pathogenic bacteria was conducted using the 3-[4,5-dimethylthiazol-2yl]-2,5-diphenyltetrazolium (MTT) reduction assay. At first, the bacterial cells (10^6^ CFU/mL) were cultured in sterile growth medium in either the presence or the absence of the test compounds (UDCA and ciprofloxacin) at 37 °C for 24 h. After incubation, the planktonic cells were removed and 20 µL MTT (A 5 mg/mL) was added to each well and incubated for 3 h at 37 °C to generate formazan. The formazan crystals were dissolved by adding 200 µL of DMSO, and the absorbance was measured at 570 nm.

### 2.14. Estimation of Lipase Production

The investigation of lipase synthesis by *S. aureus* was performed utilizing the methodology described by Lee et al. [29]. To summarize, 20 μL of *S. aureus* culture was introduced to 2 mL of brain–heart infusion medium. The mixture was then incubated for 24 h at 37 °C, either with or without the presence of UDCA–ciprofloxacin. The cultured sample was subjected to centrifugation at 8000× *g* for 10 min. Afterwards, 0.1 mL portions were combined with 0.9 mL of a mixture containing 10% (*v*/*v*) buffer A (consisting of 3 mg/mL p-nitrophenyl palmitate in isopropyl alcohol) and 90% (*v*/*v*) buffer B (consisting of 1 mg/mL gum arabic and 2 mg/mL sodium deoxycholate in Na2PO4 buffer, pH 8.0). The mixture was then heated for 30 min, in the dark, at 40 °C. The reactions were stopped by the addition of 1 M Na_2_CO_3_, and the mixtures were thereafter centrifuged at level 10, for a duration of 10 min. The optical densities were recorded at a wavelength of 405 nm.

### 2.15. Measurement of Staphyloxanthin Production

Staphyloxanthin has been identified as a possible virulence factor that facilitates the development and reproduction of *S. aureus* by modulating the host’s immune response. The production of staphyloxanthin was evaluated by assessing the quantity of the compound under the impact of various combinations of test chemicals, following the approach published by Lee et al. [29]. Concisely, 20 µL of *S. aureus* overnight culture (10^6^ CFU/mL) was introduced into 2 mL of fresh growth medium. The culture was subsequently cultured for 24 h at 37 °C in a tube with continuous agitation at a speed of 250 rpm, both with and without the presence of test chemicals. Following incubation, the culture underwent centrifugation at 8000× *g* for 10 min. Subsequently, the pellet underwent a washing process using sterile phosphate buffered saline (PBS) and was then reconstituted in pure ethanol. The reconstituted pellet was then incubated at a temperature of 40 °C for a duration of 20 min. The cells were removed by centrifuging them at 10,000× *g* for 10 min. Subsequently, the liquid’s absorbance above the sediment was quantified at a wavelength of 450 nm.

### 2.16. In Vitro Infection Studies

To evaluate the Effect of the UDCA–ciprofloxacin combinations on normal cells during bacterial infection, the normal human dermal fibroblasts (NHDF) cell line was used as per the method described by Gilchrist et al. [30], with some slight modification. The cells were seeded in a 24-well plate at a density of 10^5^ cells/mL for 12 h in RPMI 1640 medium supplemented with 10% FBS and 1% antibiotic–antimycotic solution in 5% CO2. Subsequently, the growth medium was substituted for with a fresh RPMI 1640 complete medium that lacked antibiotics. The medium was then infected with *S. aureus* at a multiplicity of infection of 1. This was performed in the presence of either the combination of UDCA–ciprofloxacin or the individual components alone for a duration of 12 h. After incubation, the media was removed, and cells were washed with PBS thrice. Subsequently, the cells were disrupted in sterile Milli-Q water and the bacteria were spread on nutritional agar to quantify the colony-forming units (CFUs), which were expressed as CFU per well.

### 2.17. Statistical Analysis

One-way ANOVA was used to assess multiple comparisons across groups using GraphPad Prism (version 9). Mean values, together with their corresponding standard deviations, were calculated from three separate experiments. *p*-values with a value less than 0.05 were considered significant.

## 3. Results

### 3.1. MIC and FICI Determination

**Figure 1:**
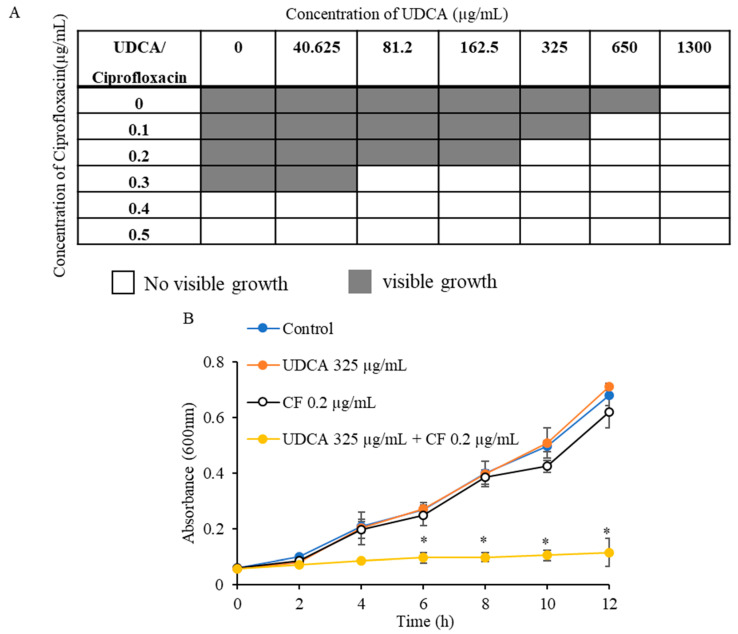
Combined effects of UDCA and ciprofloxacin on *S. aureus*. Checkerboard analysis of combined treatment of UDCA and ciprofloxacin-A: Effect of combined action of UDCA–ciprofloxacin on planktonic cells-D. UDCA—ursodeoxycholic acid, CF—ciprofloxacin. * *p* < 0.05, significant in comparison to control.

In this study, the MIC of UDCA was assessed via the broth microdilution method. MIC is the lowest concentration of any given compound which can inhibit the growth of a microorganism. The MIC of UDCA was assessed to be 1300 µg/mL against *S. aureus* ATCC 25923 (Table 1). As the experiment was designed to study the synergistic effect of UDCA with ciprofloxacin, the MIC of ciprofloxacin was also analyzed against *S. aureus*. The MIC of the antibiotic ciprofloxacin was found to be 0.5 µg/mL (Table 1). In addition, a checkerboard method was employed to assess the effectiveness of the merging of ciprofloxacin and UDCA in eradicating *S. aureus* biofilm. The checkerboard assay illustrated that when a sub-MIC concentrationof UDCA (325 µg/mL) was combined with a sub-MIC concentrationof ciprofloxacin (0.2 µg/mL), an efficient inhibition in the growth of *S. aureus* was observed (Figure 1A). The experiment also indicated a 4-fold reduction in the MIC of UDCA in combination with ciprofloxacin. Likewise, the MIC of ciprofloxacin was reduced to 0.2 µg/mL in combination analysis. The results of checkerboard assay showed that the FIC index for UDCA–ciprofloxacin against *S. aureus* was found to be 0.65. The results also indicated that UDCA concentration lower than 325 µg/mL didn’t affect microbial growth. To further verify, we studied the growth pattern of *S. aureus* in the absence or presence of a combination of UDCA–ciprofloxacin. The observation indicated that UDCA alone, at a dose of 325 µg/mL, did not show any significant alterations in the pattern of microbial growth (Figure 1B). The presence of ciprofloxacin at a concentration of 0.2 µg/mL did not have any effect on the pattern of microbial development, as shown in Figure 1B. Nevertheless, the development of *S. aureus* was significantly diminished when the bacteria were subjected to incubation with both UDCA (325 µg/mL) and ciprofloxacin (0.2 µg/mL) (Figure 1B).

### 3.2. Action against Biofilm

**Figure 2:**
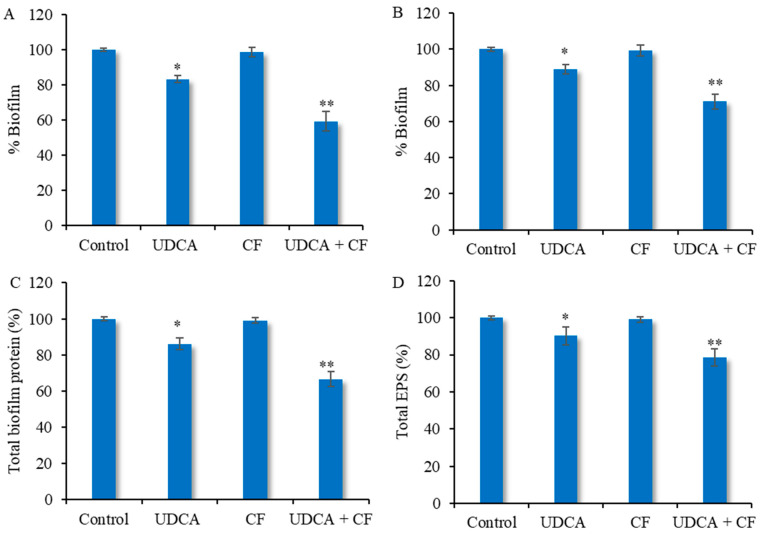
Effect of UDCA in combination with ciprofloxacin on *S. aureus* biofilm: (**A**)—effect on biofilm formation, (**B**)—effect on biofilm eradication, (**C**)—effect on total biofilm protein, and (**D**)—effect on biofilm EPS. UDCA—ursodeoxycholic acid (325 µg/mL), CF—ciprofloxacin (0.2 µg/mL). Data presented as mean ± SD. * *p* < 0.05, significant in comparison to control. ** *p* < 0.01, significant in comparison to control.

**Figure 3:**
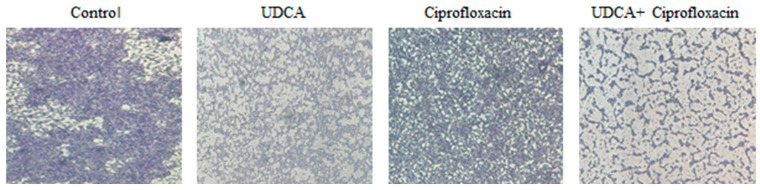
Effect of different treatments on *S. aureus* biofilm structure formed on glass coverslips stained with crystal violet. Image taken at 200× magnification.

The primary objective of this study was to create and execute a combined approach utilizing UDCA and ciprofloxacin to target biofilms. In order to enhance the antibiofilm impact, we made our decision based on the outcome of the checkerboard analysis. The dose of UDCA opted for was 325 µg/mL and that of ciprofloxacin was 0.2 µg/mL, which was the optimal combination strategy determined by the FIC assessment. The combination, along with the individual compounds, was analyzed as to the inhibition of the biofilm-forming capability of *S. aureus*. Treatment with UDCA or ciprofloxacin alone led to approximately 16.75% and 1.27% biofilm inhibition levels, respectively. The results indicated a minimum of biofilm formation by *S. aureus* after 24 h of incubation when treated with the combination (40.64% biofilm inhibition) (Figure 2A). A similar effect was also observed when change in biofilm structure/density was evaluated. The reduced density of cells in biofilm observed on the crystal violet-stained coverslips in the dual treatment group was superior to those of either of the agents alone (Figure 3). Additionally, the dual combination was also assessed for eradication of pre-formed biofilm (Figure 2B). Similarly, the use of UDCA alone resulted in a minor reduction of around 11.9% in biofilm formation compared to the untreated group, while no significant effect was observed in the ciprofloxacin-treated group. Likewise, the combination treatment exhibited a significant reduction in the amount of biofilm, as assessed via the crystal violet method (28.94% biofilm reduction). However, it was also observed that the dual combination acted in a comparatively more efficient manner in biofilm inhibition relative to the biofilm eradication.

### 3.3. Effect on Biofilm Protein and EPS

In order to measure the amount of protein in the biofilm, the total protein content in the biofilm mass was calculated for both the treated and untreated circumstances. Figure 2C shows that the bacteria produced the most biofilm protein when no test agents were used. An apparent reduction of 14% in biofilm protein was noted when the bacterial cells were treated only with UDCA. However no significant reduction in biofilm protein was seen when the cells were treated with ciprofloxacin alone. When the cells were treated with the combination of test compounds (UDCA–ciprofloxacin), the biofilm protein content was decreased to 66.65% in comparison to control (Figure 2C). In line with the findings from prior observations, the impact on EPS generation, relative to whether the test substances were present or not, exhibited comparable trends. The untreated set showed the highest quantity of EPS (100%) (Figure 2D). EPS production in the UDCA and ciprofloxacin-treated groups exhibited levels of 90.2% and 99.1%, respectively. Nevertheless, the synthesis of EPS was significantly decreased when the combination of UDCA and ciprofloxacin was used (78.56%) (Figure 2D), resulting in a detrimental impact on colonization.

### 3.4. Effect of Combined Treatment on Underlying Mechanisms

**Figure 4:**
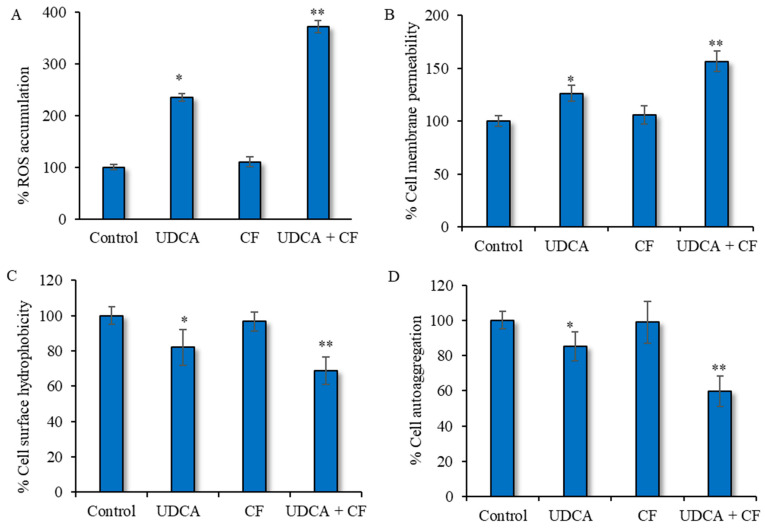
Combined effects of UDCA and ciprofloxacin treatment on cellular ROS profile—(**A**), on bacterial cell permeability—(**B**), on cell surface hydrophobicity—(**C**), and on cell auto-aggregation—(**D**) in *S. aureus* biofilm. UDCA—ursodeoxycholic acid (325 µg/mL), CF—ciprofloxacin (0.2 µg/mL). Data presented as mean ± SD. * *p* < 0.05, significant in comparison to control. ** *p* < 0.01, significant in comparison to control.

This experiment entailed quantifying the amounts of ROS in *S. aureus* cells that were exposed to a combination of UDCA and ciprofloxacin, as well as those exposed to each component separately. When the bacterial cells were exposed to ciprofloxacin alone, there was no notable alteration in the formation of ROS compared to the untreated group. However, treatment with UDCA alone was able to cause a significant (*p* < 0.05) increase in ROS levels. The findings also indicated a noteworthy (*p* < 0.01) rise in ROS generation when cells were given UDCA along with ciprofloxacin (Figure 4A), establishing this approach as the most effective treatment. A similar pattern was observed when the treated microbial cells were analyzed for any change in cell membrane permeability. The results demonstrated that UDCA treatment significantly (*p* < 0.01) elevated the membrane permeability of microbial cells while ciprofloxacin alone did not initiate any significant change in cell membrane permeability (Figure 4B). The co-treatment with UDCA–ciprofloxacin caused a 76% rise in membrane permeability in *S. aureus* cells, demonstrating the effectiveness of the combination in improving membrane permeability (Figure 4B). In terms of individual components, only UDCA (325 μg/mL) was able to significantly (*p* < 0.01) increase the cellular permeability. Furthermore, *S. aureus* surface hydrophobicity was assessed in various treatment scenarios, as it plays a significant role in microbial adhesion [5]. Our analysis found that the surface hydrophobicity of *S. aureus* cells was reduced by around 17.94% in the group treated with UDCA. The group treated with ciprofloxacin alone didn’t show a significant change in cell surface hydrophobicity. A superior effect was observed when bacterial cells were treated with the UDCA–ciprofloxacin combination, revealing a significant (*p* < 0.01) 39% reduction in cell surface hydrophobicity, which impeded the attachment of the microbial cells to the substratum (Figure 4C). Further, the test-compound combination was evaluated for cellular auto-aggregation. Our observation showed that the cellular auto-aggregation decreased by approximately 1.67 times when the cells were given UDCA in combination with ciprofloxacin (Figure 4D). In a finding similar to previous results, only UDCA alone significantly (*p* < 0.05) reduced the cellular auto-aggregation.

### 3.5. Effect on Bacterial Metabolic Activity

**Figure 5:**
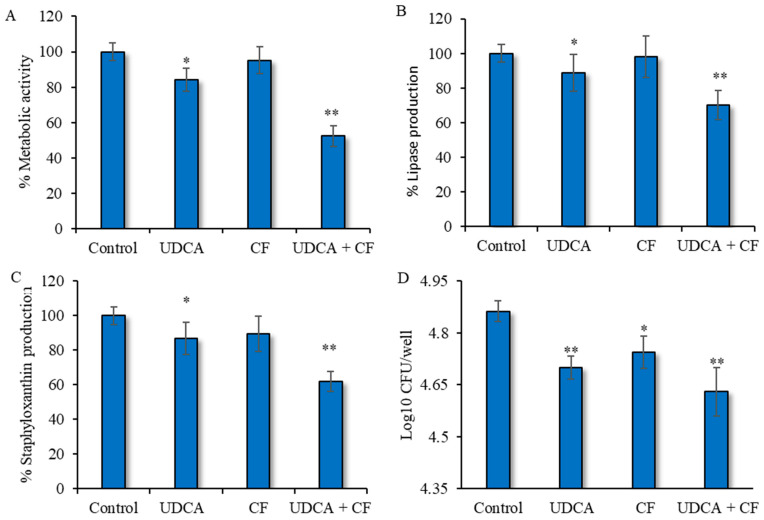
The effect of UDCA in combination with ciprofloxacin on metabolic activity—(**A**), and on virulence factors/lipase production—(**B**), staphyloxanthin production in *S. aureus*—(**C**), and effect on bacterial killing within NHDF cells. CFU is quantified from internalized microbe within macrophage. UDCA—ursodeoxycholic acid (325 µg/mL), CF—ciprofloxacin (0.2 µg/mL). Data presented as mean ± SD. * *p*< 0.05, significant in comparison to control. ** *p* < 0.01, significant in comparison to control.

The increase in biofilm-related infections can be attributed to the increased abundance of cells that are metabolically active Therefore, the MTT test was conducted to measure the quantity of metabolically active cells present in the biofilm. The conversion of MTT into formazan is directly contingent upon the respiratory activity and is positively associated with the cellular viability. The metabolic activity of the adherent microorganisms decreased by approximately 16% when supplied with UDCA alone, as seen in Figure 5A. No significant alteration in the metabolic activity of microbes was observed under the influence of ciprofloxacin alone. However, when the combination of UDCA and ciprofloxacin was applied, the metabolic activity was found to be significantly (*p* < 0.01) reduced. The lower metabolic activity indicated the low viability of the bacteria in the combination treatment.

### 3.6. Effect on Virulence Factors of S. aureus

We conducted an experiment to determine the impact on the release of virulence factors from *S. aureus* after being exposed to the simultaneous treatment of the test compounds. The experiment showed a notable decline in lipase synthesis, with a reduction of around 12.2%, when *S. aureus* biofilm cells were exposed to UDCA only. However, ciprofloxacin alone was not able to restrict lipase production in *S. aureus*. When bacterial cells were treated with the UDCA–ciprofloxacin combination, lipase production was reduced to approximately 30% (Figure 5B). Production of another virulence factor, staphyloxanthin, was also evaluated under different treatment conditions. The observations showed that UDCA alone significantly reduced staphyxanthin synthesis (*p* < 0.01). However, when ciprofloxacin was combined with UDCA, the chosen dosage resulted in the most effective diminution, at around 38%, in the formation of staphyloxanthin, as shown in Figure 5C. Overall, the observations suggested that the combination of the chosen doses of the chemicals effectively decreased the release of many virulence factors from *S. aureus*.

### 3.7. Effect of the UDCA–Ciprofloxacin Combination on S. aureus Intracellular Replication within Normal NHDF Cells

In this experiment, we tried to evaluate whether UDCA in conjugation with ciprofloxacin could restrict *S. aureus* replication within NHDF cells. The cells were treated with the different test compounds, either alone or in combination, and the results were presented as the number of CFU erupted at 24 h post infection. Replication of *S. aureus* was significantly reduced in comparison to the untreated control in all the treated groups, as quantified via CFUs (*p* < 0.01, Figure 5D). Among all the treated groups, the UDCA–ciprofloxacin treatment exhibited the maximum efficiency in reducing CFUs. Nevertheless, no significant difference was identified among any of the treated groups.

## 4. Discussion

*S. aureus* is one of the major pathogens capable of producing biofilm. In addition to providing resistance to antibiotics, biofilms also contribute significantly to the advancement of chronic illnesses. After the formation of a biofilm, individual cells have the ability to separate from the initial biofilm and either initiate additional sites of infection or facilitate a sudden infection like sepsis [31]. Thus, this generates a pressing demand for innovative approaches to combat illnesses linked with biofilms. In search of new compounds, we previously explored the action of ursodeoxycholic acid and found a positive effect in combating sessile pathogenic microbes [18]. Thus, the current study explored the antimicrobial and antibiofilm activities of UDCA, particularly in combination with an antibiotic, ciprofloxacin, against *S. aureus* ATCC 25923, in order to analyze the synergistic effect, if any.

The concurrent use of UDCA and ciprofloxacin exhibited an approximate synergistic impact against *S. aureus*, exhibiting a 0.65 FIC index. This synergy resulted in 4-fold and 2.5-fold reductions in the MIC of UDCA and ciprofloxacin, respectively, when used in combination, suggesting enhanced antibacterial efficacy at lower doses. This synergy is crucial, as it not only enhances the antibacterial efficacy but also suggests a potential for overcoming antibiotic resistance. Similar synergy was also observed between UDCA and colistin, in which the combined treatment reduced the MIC of colistin-resistant strains 4–64 times [32]. This combination of UDCA and ciprofloxacin has shown significant effectiveness in both preventing the formation of biofilms and eliminating pre-existing biofilms of *S. aureus*. This dual action is especially noteworthy because biofilms are notoriously resistant to treatment and frequently cause persistent illnesses. However, the combination was more effective in inhibiting biofilm formation rather than eradicating existing biofilms, as evidenced by the crystal violet assay results. This could be due to the improved ability to obstruct the initial phases of biofilm development shown by the test compounds. Prior research has also demonstrated that the combination of zingerone and ciprofloxacin has a more significant effect in inhibiting the development of biofilms compared to entirely eradicating pre-existing biofilms [33]. Moreover, a combination therapy with a synergistic impact can significantly contribute to addressing antibiotic resistance. An established example of this strategy is evident in tuberculosis treatment, in which the co-administration of streptomycin and para-aminosalicylic acid has resulted in successful management of resistant bacteria [34]. Furthermore, this combination therapy has contributed to the mitigation of resistance development, resulting in a superior outcome [35].

Antibiofilm agents employ diverse strategies to inhibit the formation of biofilms. Oxidative stress caused by ROS in microbial cells can be a deliberate tactic that causes cellular damage and disrupts the architecture of biofilms [36]. Concomitant administration of UDCA and ciprofloxacin was observed to markedly enhance the generation of ROS, resulting in heightened oxidative stress inside the microbial cells. Furthermore, the combined therapy led to an augmentation in cellular permeability and a modification in the hydrophobicity of the cell surface, a critical factor for microbial adherence. Prior research has indicated that a decrease in the hydrophobicity of the cell surface can have a negative impact on the production of *S. aureus* biofilms [37]. The hydrophobic properties of microbial surfaces enhance their capacity to attach to both inanimate and animate surfaces, as well as to penetrate host tissues. Our analysis found that the cell surface hydrophobicity of treated cells fell by over 1.5 times, which greatly hindered the attachment of microbial cells to the substratum. According to the literature, the hydrophobicity of the cell surface can affect how cells stick together, which in turn increases their ability to cling to surfaces [38]. The results of our observation showed a substantial reduction of around 44% in cellular auto-aggregation when the cells were exposed to a combination of UDCA and ciprofloxacin. Therefore, the findings indicated that cellular auto-aggregation and cell surface hydrophobicity exhibited a parallel pattern when influenced by the compounds, regardless of whether they were employed individually or in conjunction. The data collectively demonstrated that co-treatment with the test compounds impeded the hydrophobicity of the cell surface, leading to a reduction in cellular auto-aggregation and impacting the formation of microbial biofilms.

The combined administration of UDCA and ciprofloxacin resulted in a notable decrease in the metabolic activity of *S. aureus*, as evidenced by the MTT assay. The combined treatment is further demonstrated to be beneficial by the correlation between reduced bacterial viability and lower metabolic activity. Furthermore, when considering the mechanism by which the combination of UDCA and ciprofloxacin affects *S. aureus* biofilm, it should be noted that the production of EPS plays a crucial role. EPS, consisting of proteins embedded inside it, has a vital function in establishing the shape of biofilms. It provides protection against unfavorable environmental conditions and contributes to the viscoelastic properties of biofilms [10]. The combination therapy led to a reduction of approximately 1.5 times in the amount of protein in the biofilm and a significant drop in the synthesis of EPS. The alteration in the structure of biofilm emphasizes the capability of UDCA and ciprofloxacin to diminish the protective properties of biofilm and enhance the effectiveness of antimicrobial treatment. The presence of these components is essential for the construction and function of biofilms, and their decrease implies a significant anti-biofilm impact. Moreover, the virulence factors produced by *S. aureus* are essential for the formation of biofilms and for evading the host’s innate immune response. Staphyloxanthin provides protection against phagocytosis in *S. aureus* and enhances the stability of the bacterial cell membrane by arranging the alkyl chains of lipids in the membrane, which ultimately prolongs the longevity of *S. aureus*. In the same way, it has been shown that lipase produced by *S. aureus* disrupts the function of the host’s granulocytes and enhances the bacteria’s ability to survive throughout the host’s defense by deactivating the bacteriocidal lipids [39]. UDCA–ciprofloxacin successfully prevented the formation of biofilms by suppressing the production of virulence factors, including staphyloxanthin and lipase. The study found that UDCA–ciprofloxacin increased autophagy in NHDF cells and limited the growth of microorganisms. This discovery implies that the combination therapy may have a notable efficacy in addressing intracellular infections, which are frequently difficult to manage solely with traditional antibiotics.

The results concerning the synergy of UDCA with ciprofloxacin against *S. aureus* biofilms bear considerable relevance for practical therapeutic approaches. Contemporary approaches for addressing biofilm-associated infections focus on inhibiting biofilm formation, restricting biofilm growth, or eradicating biofilms using chemical or mechanical interventions (e.g., removal) [40,41,42]. UDCA as an additional therapy may augment the efficacy of ciprofloxacin in addressing persistent infections linked to biofilms, which are notoriously challenging to eliminate. As this combination treatment altered cell membrane permeability and surface hydrophobicity, this could facilitate greater antibiotic penetration into bacterial cells, further enhancing the effectiveness of ciprofloxacin. This combination therapy may enable reduced dosages of each medication, potentially decreasing side effects and toxicity while simultaneously enhancing the therapeutic efficacy [43]. In past, many researchers have exploited this approach for combating biofilm formation via in vitro studies [44,45,46]. This is especially crucial in addressing infections caused by antibiotic-resistant forms of *S. aureus*, necessitating novel treatment strategies. Considering the ubiquity of biofilms in chronic wounds [4], the UDCA–ciprofloxacin combination may prove especially advantageous in wound care environments, where recurrent infections frequently arise following conventional therapies [47,48,49]. Moreover, bile salts enhance the epithelial transport of hydrophilic drugs through the paracellular pathway and improve the movement of hydrophobic compounds via both paracellular and transcellular pathways, thereby offering additional benefits [50]. The study concentrated on *S. aureus*, although the processes via which UDCA improves antibiotic effectiveness may also apply to other biofilm-forming bacteria, indicating that this combination warrants investigation for the treatment of a broader spectrum of diseases. These findings establish a basis for additional study into UDCA’s function as an adjuvant therapy in antibiotic treatments. This research might require further pre-clinical and clinical investigations to confirm the effectiveness of UDCA across a variety of populations.

## 5. Conclusions

*S. aureus* biofilms pose a significant threat to worldwide public health, primarily because of their escalating drug resistance. Considering the extensive prevalence of *S. aureus* biofilm development, it is imperative to devise a strong approach to address this problem. Our research signifies a noteworthy advancement in the management of *S. aureus* infections. We have proven that the combination of ursodeoxycholic acid (UDCA) and ciprofloxacin efficiently prevents the production of *S. aureus* biofilms and breaks down already-formed biofilms. In addition, our experimental results demonstrate that this combination therapy decreases the release of different *S. aureus* virulence factors. This novel strategy presents a hopeful solution to the current worldwide problems caused by *S. aureus* infections, potentially transforming treatment methods and enhancing patient results on a global scale.

## Figures and Tables

**Table 1:** MICs of the test compounds.

Test Compound	*S. aureus* ATCC 25923
	MIC (µg/mL)
UDCA	1300
Ciprofloxacin	0.5

## Data Availability

The original contributions presented in the study are included in the article, further inquiries can be directed to the corresponding authors.
